# Predictive factors and outcomes for ibrutinib in relapsed/refractory marginal zone lymphoma: a multicenter cohort study

**DOI:** 10.1186/s13045-022-01316-1

**Published:** 2022-07-16

**Authors:** Narendranath Epperla, Qiuhong Zhao, Sayan Mullick Chowdhury, Lauren Shea, Tamara K. Moyo, Nishitha Reddy, Julia Sheets, David M. Weiner, Praveen Ramakrishnan Geethakumari, Malathi Kandarpa, Ximena Jordan Bruno, Colin Thomas, Michael C. Churnetski, Andrew Hsu, Luke Zurbriggen, Cherie Tan, Kathryn Lindsey, Joseph Maakaron, Paolo F. Caimi, Pallawi Torka, Celeste Bello, Sabarish Ayyappan, Reem Karmali, Seo-Hyun Kim, Anna Kress, Shalin Kothari, Yazeed Sawalha, Beth Christian, Kevin A. David, Irl Brian Greenwell, Murali Janakiram, Vaishalee P. Kenkre, Adam J. Olszewski, Jonathon B. Cohen, Neil Palmisiano, Elvira Umyarova, Ryan A. Wilcox, Farrukh T. Awan, Juan Pablo Alderuccio, Stefan K. Barta, Natalie S. Grover, Nilanjan Ghosh, Nancy L. Bartlett, Alex F. Herrera, Geoffrey Shouse

**Affiliations:** 1 Division of Hematology, Department of Medicine, The Ohio State University, Columbus, OH 43210 USA; 2 Washington University, St. Louis, MO USA; 3 Levine Cancer Center, Atrium Health, Charlotte, NC USA; 4 Vanderbilt University Medical Center, Nashville, TN USA; 5 University of North Carolina, Chapel Hill, NC USA; 6 Perelman School of Medicine at the University of Pennsylvania, Philadelphia, PA USA; 7 Harold C. Simmons Comprehensive Cancer Center, University of Texas Southwestern Medical Center, Dallas, TX USA; 8 Rogel Cancer Center, University of Michigan, Ann Arbor, MI USA; 9 University of Vermont, Burlington, VT USA; 10 Thomas Jefferson University, Philadelphia, PA USA; 11 Winship Cancer Institute, Emory University Medical Center, Atlanta, GA USA; 12 Brown University, Providence, RI USA; 13 University of Wisconsin, Madison, WI USA; 14 Cancer Institute of New Jersey, New Brunswick, NJ USA; 15 Medical University of South Carolina, Charleston, SC USA; 16 University of Minnesota, Minneapolis, MN USA; 17 University Hospitals Seidman Cancer Center, Cleveland, OH USA; 18 Roswell Park Cancer Institute, Buffalo, NY USA; 19 H. Lee Moffitt Cancer Center and Research Institute, Tampa, FL USA; 20 University of Iowa, Iowa City, IA USA; 21 Northwestern University, Chicago, IL USA; 22 Rush University, Chicago, IL USA; 23 Yale University, New Haven, CT USA; 24 University of Miami, Miami, FL USA; 25 City of Hope, Duarte, CA USA

**Keywords:** Marginal zone lymphoma, MZL, Ibrutinib, Relapsed, Refractory

## Abstract

Ibrutinib is effective in the treatment of relapsed/refractory (R/R) marginal zone lymphoma (MZL) with an overall response rate (ORR) of 48%. However, factors associated with response (or lack thereof) to ibrutinib in R/R MZL in clinical practice are largely unknown. To answer this question, we performed a multicenter (25 US centers) cohort study and divided the study population into three groups: “ibrutinib responders”—patients who achieved complete or partial response (CR/PR) to ibrutinib; “stable disease (SD)”; and “primary progressors (PP)”—patients with progression of disease as their best response to ibrutinib. One hundred and nineteen patients met the eligibility criteria with 58%/17% ORR/CR, 29% with SD, and 13% with PP. The median PFS and OS were 29 and 71.4 months, respectively, with no difference in PFS or OS based on the ibrutinib line of therapy or type of therapy before ibrutinib. Patients with complex cytogenetics had an inferior PFS (HR = 3.08, 95% CI 1.23–7.67, *p* = 0.02), while those with both complex cytogenetics (HR = 3.00, 95% CI 1.03–8.68, *p* = 0.04) and PP (HR = 13.94, 95% CI 5.17–37.62, *p* < 0.001) had inferior OS. Only primary refractory disease to first-line therapy predicted a higher probability of PP to ibrutinib (RR = 3.77, 95% CI 1.15–12.33, *p* = 0.03). In this largest study to date evaluating outcomes of R/R MZL treated with ibrutinib, we show that patients with primary refractory disease and those with PP on ibrutinib are very high-risk subsets and need to be prioritized for experimental therapies.

**Supplementary Information:**

The online version contains supplementary material available at 10.1186/s13045-022-01316-1.

## To the editor

Marginal zone lymphomas (MZL) are the third most common B cell non-Hodgkin lymphoma (NHL) comprising 7% of all NHLs [1–3]. Ibrutinib was FDA-approved for relapsed or refractory (R/R) MZL based on phase II clinical trial that showed an overall response rate (ORR) of 48% [4]. In the recently updated long-term follow-up of this study, the ORR was 58% with a median duration of response (DOR) of 27.6 months [5]. However, factors associated with response (or lack thereof) to ibrutinib in R/R MZL in clinical practice are largely unknown. Hence, we sought to evaluate characteristics predictive of ibrutinib failure in R/R MZL and describe the outcomes of patients on ibrutinib therapy in a “*real-world”* setting.

In this multicenter retrospective cohort study, we included adult patients (18 years or older) with R/R MZL who received ibrutinib monotherapy between 2010 and 2019 at 25 US medical centers. The study population was divided into 3 groups: "ibrutinib responders”—patients who achieved a complete response (CR) or partial response (PR) to ibrutinib as their best response; “stable disease (SD)”; and “primary progressors (PP)”—patients with progression of disease as their best response to ibrutinib. The primary objective of the study was to evaluate the *real-world* efficacy outcomes of ibrutinib in R/R MZL including response rates, duration of response (DOR), progression-free survival (PFS), and overall survival (OS). Secondary objectives included the evaluation of factors predictive of PP, PFS, and OS. See supplementary appendix for definitions and statistical analysis.

A total of 119 patients met the inclusion criteria. Sixty-nine patients achieved a disease response (ORR 58% with a CR rate of 17%), 35 (29%) had SD, and 15 (13%) had PP. Table 1 shows the baseline characteristics of the patient population. Among the 69 patients who achieved CR/PR, median DOR was 36.8 months (95% CI 25.5-NR) (Additional file 1: Fig. S1A). When stratified by CR or PR status (Additional file 1: Fig. S1B), median DOR was not reached (NR) (95% CI 32-NR) in patients who achieved CR compared to 26 months (95% CI 20.2-NR) in those achieving PR (*p* = 0.057). Median PFS and OS for the entire group (*n* = 119) were 29 months (Additional file 1: Fig. S2A) and 71.4 months (Additional file 1: Fig. S2B), respectively. The 1-year and 2-year PFS and OS rates were 66% and 55%, and 87% and 85%, respectively. When stratified by the ibrutinib line of therapy (second line vs. third line vs. fourth line and beyond), there was no difference in PFS (median PFS in similar order, 28.5 months vs. 28.2 months vs. 39.8 months, respectively, *p* = 0.89, Additional file 1: Fig. S3A) or OS (median OS in similar order, NR vs. 71.4 months vs. 44.5 months, respectively, *p* = 0.37, Additional file 1: Fig. S3B). Among the factors evaluated to determine the predictors of PFS and OS (see Additional file 1: Tables S1 and S2), complex cytogenetics portended inferior PFS (HR = 3.08, 95% CI 1.23–7.67, *p* = 0.02), while both complex cytogenetics (HR = 3.00, 95% CI 1.03–8.68, *p* = 0.04) and PP (HR = 13.94, 95% CI 5.17–37.62, *p* < 0.001) were associated with poor OS. Among the factors evaluated for association with PP (Table 2), only primary refractory disease (to first-line therapy) predicted a higher probability of PP to ibrutinib (RR = 3.77, 95% CI 1.15–12.33, *p* = 0.03). Lastly, the prior line of therapy (Additional file 1: Table S3) was not associated with differences in outcomes associated with ibrutinib therapy (Additional file 1: Figs. S4 and S5).

**Table 1:** Baseline characteristics

	All patients *N* = 119 (%)	IB CR + PR *N* = 69 (%)	IB SD * N* = 35 (%)	IB PD* N* = 15 (%)	*p* value
Median age at diagnosis in years (range)	64 (23–90)	66 (23–90)	63 (40–86)	64 (38–89)	0.67
Median age at ibrutinib therapy in years (range)	68 (27–91)	69 (27–90)	67 (42–86)	65 (41–91)	0.74
Gender					0.89
Male	55 (46)	33 (48)	15 (43)	7 (47)	
Female	64 (54)	36 (52)	20 (57)	8 (53)	
BMI					0.95
< 30	71 (71)	43 (72)	20 (69)	8 (73)	
≥ 30	29 (29)	17 (28)	9 (31)	3 (27)	
Missing	19	9	6	4	
ECOG PS at diagnosis					0.53
0	46 (46.5)	25 (42)	13 (50)	8 (61)	
1	47 (47.5)	32 (53)	11 (42)	4 (31)	
≥ 2	6 (6)	3 (5)	2 (8)	1 (8)	
Missing	20	9	9	2	
MZL subtype					0.97
NMZL	50 (42)	28 (41)	17 (49)	5 (33)	
SMZL	29 (24)	17 (25)	8 (23)	4 (27)	
EMZL	40 (34)	24 (34)	10 (28)	6 (40)	
Stage at diagnosis					0.95
1–2	19 (17)	11 (16)	6 (18)	2 (14)	
3–4	96 (83)	56 (84)	28 (82)	12 (86)	
Missing	4	2	1	1	
B symptoms at diagnosis					0.62
No	81 (74)	44 (70)	25 (78)	12 (80)	
Yes	29 (26)	19 (30)	7 (22)	3 (20)	
Missing	9	6	3	0	
LDH higher than institutional baseline					0.80
No	70 (71)	43 (71)	20 (74)	7 (64)	
Yes	29 (29)	18 (29)	7 (26)	4 (36)	
Missing	20	8	8	4	
Albumin at diagnosis					0.75
Normal	80 (81)	49 (80)	22	9	
Low	19 (19)	12 (20)	4	3	
Missing	20	8	9	3	
Monoclonal protein at diagnosis					0.05
No	49 (56)	30 (54)	17 (74)	2 (25)	
Yes	38 (44)	26 (46)	6 (26)	6 (75)	
Missing	32	13	12	7	
BM involvement at diagnosis					0.72
No	32 (32)	17 (30)	10 (33)	5 (42)	
Yes	67 (68)	40 (70)	20 (67)	7 (58)	
Not done	20	11	5	3	
*TP53* mutation/17p deletion (*n* = 67)*					0.99
No	19 (28)	10 (23)	7 (47)	2 (25)	
Yes	10 (15)	7 (16)	2 (13)	1 (13)	
Unavailable/not tested	38 (57)	27 (61)	6 (40)	5 (62)	
Complex cytogenetics (*n* = 67)*					0.16
No	57 (85)	37 (90)	17 (85)	6 (67)	
Yes	10 (15)	4 (10)	3 (15)	3 (33)	
Primary refractory disease**					0.07
No	89 (75)	56 (81)	25 (71)	8 (53)	
Yes	30 (25)	13 (19)	10 (29)	7 (47)	
First-line therapy					0.47
Rituximab	58 (49)	35 (51)	19 (54)	4 (27)	
BR	30 (25)	16 (23)	9 (26)	5 (33)	
R-CVP	11 (9)	7 (10)	1 (3)	3 (20)	
R-CHOP	9 (8)	5 (7)	2 (6)	2 (13)	
Others	11 (9)	6 (9)	4 (11)	1 (7)	
Receipt of maintenance R					0.27
No	88 (74)	47 (68)	29 (83)	12 (80)	
Yes	31 (26)	22 (32)	6 (17)	3 (20)	
Line of ibrutinib therapy					0.40
Second line	54 (45)	31 (45)	16 (46)	7 (47)	
Third line	41 (35)	27 (39)	9 (26)	5 (33)	
Fourth line and beyond	24 (20)	11 (16)	10 (28)	3 (20)	
Median f/up in months (range)^	23 (1–75)	23 (1–72)	26 (3–75)	6 (3–22)	

*CR* complete response, *PR* partial response, *SD* stable disease, *PD* progressive disease, *BMI* body mass index, *ECOG PS* Eastern Cooperative Oncology Group performance status, *MZL* marginal zone lymphoma, *LDH* lactate dehydrogenase, *BM* bone marrow, *BR* bendamustine rituximab, *R-CVP* rituximab, cyclophosphamide, vincristine, prednisone, *R-CHOP* rituximab, cyclophosphamide, Adriamycin, vincristine, prednisone, *f/up* follow-up

*Only among those who had bone marrow involvement. Complex karyotype was defined as the presence of at least three chromosomal aberrations in at least two cells

**Primary refractory disease: defined as progression of disease at the end of induction therapy or within 6 months of treatment completion. Among these 30 patients, 13 received rituximab, 9 received BR, 4 received R-CHOP, 3 received R-CVP, 1 received other

^Among those who are alive

**Table 2:** Modeling on risk of progression on ibrutinib

Variable	PP versus CR/PR			SD versus CR/PR		
	RR	95% CI	*p* value	RR	95% CI	*p* value
Age at diagnosis	1.02	0.96–1.08	0.61	0.99	0.96–1.03	0.65
Gender						
Male	Referent					
Female	1.05	0.34–3.22	0.93	1.22	0.54–2.78	0.63
BMI						
< 30	Referent					
≥ 30	0.95	0.22–4.04	0.94	1.14	0.43–3.01	0.79
ECOG PS at diagnosis						
0	Referent					
1	0.39	0.10–1.46	0.16	0.66	0.25–1.73	0.40
≥ 2	1.04	0.09–11.61	0.97	1.28	0.19–8.75	0.80
MZL subtype						
NMZL	Referent					
SMZL	1.32	0.31–5.63	0.71	0.78	0.27–2.19	0.63
EMZL	1.40	0.38–5.20	0.61	0.69	0.26–1.79	0.44
Stage at diagnosis						
1–2	Referent					
3–4	1.18	0.23–6.06	0.84	0.92	0.31–2.75	0.88
B symptoms at diagnosis						
No	Referent					
Yes	0.58	0.15–2.30	0.44	0.65	0.24–1.76	0.40
LDH higher than institutional baseline						
No	Referent					
Yes	1.37	0.35–5.28	0.65	0.84	0.30–2.33	0.73
Monoclonal protein at diagnosis						
No	Referent					
Yes	3.46	0.64–18.84	0.15	0.41	0.14–1.19	0.10
BM involvement at diagnosis						
No	Referent					
Yes	0.60	0.16–2.15	0.43	0.85	0.33–2.20	0.74
*TP53* mutation/17p deletion						
No						
Yes	0.76	0.08–7.25	0.81	0.81	0.15–4.48	0.81
Complex cytogenetics						
No						
Yes	4.63	0.81–26.35	0.08	1.63	0.32–8.21	0.55
Primary refractory disease*						
No						
Yes	3.77	1.15–12.33	**0.03**	1.72	0.66–4.47	0.26
Line of ibrutinib therapy						
Second line						
Third line	0.82	0.23–2.90	0.76	0.65	0.24–1.70	0.38
Fourth line and beyond	1.21	0.26–5.54	0.81	1.76	0.62–5.04	0.29

*CR* complete response, *PR* partial response, *SD* stable disease, *PD* progressive disease, *BMI* body mass index, *ECOG PS* Eastern Cooperative Oncology Group performance status, *MZL* marginal zone lymphoma, *LDH* lactate dehydrogenase, *BM* bone marrow

*Primary refractory disease: defined as progression of disease at the end of induction therapy or within 6 months of treatment completion

In this multicenter retrospective study, we made several important observations. First, the ORR to ibrutinib was 58% with predominantly PRs (41%), which is in line with the results of the phase 2 registration trial [4, 5]. Second, the median DOR was 36.8 months and was longer in those achieving CR compared to PR (although not statistically significant). Third, patients with primary refractory disease had a significantly higher probability of progression on ibrutinib. Fourth, there was no difference in the PFS, or OS based on the number or type of prior therapies. This is in contrast to the data in mantle cell lymphoma, wherein the greatest benefit from ibrutinib was noted in patients receiving ibrutinib in earlier lines of therapy (especially second-line therapy) [6]. Fifth, the presence of complex cytogenetics was predictive of inferior PFS and OS.

The ORR and DOR with ibrutinib in R/R MZL patients in our study were in line with the results of the phase 2 registration trial [4, 5]. The median PFS, however, was longer in the current study (29 months) compared to the previously published results (15.7 months) [5]. One plausible explanation could be the receipt of rituximab monotherapy prior to ibrutinib, which was higher in the current study (49% vs. 27% in the phase 2 trial), as the median PFS was 30.4 months in the recipients of rituximab monotherapy in the clinical trial [5]. Another possible explanation is that in clinical trials routine scans are performed at frequent intervals and radiologic but asymptomatic relapses are picked up. Scheduled surveillance scans are typically less frequent outside of a clinical trial, and as a result, asymptomatic progressions may not be identified until a patient experiences clinical evidence of progression, thus making the PFS appear longer.

We did not capture the information on the toxicity and dose modification of ibrutinib (dose interruption or discontinuation) in the current study. Other limitations include the lack of data on CD5, Ki-67 expression, and *MYD88* mutation status precluding our ability to study the impact of these variables on response and survival.

In conclusion, in this first and the largest study to date to report the *real-world* outcomes of R/R MZL treated with ibrutinib, we show that patients with primary refractory disease and those with PP on ibrutinib are very high-risk subsets and need to be prioritized for experimental and cellular therapies.

## Supplementary Information

**Additional file 1**. Supplemental Appendix.

## Data Availability

Please contact author for data request.

## Abbreviations

MZL: Marginal zone lymphoma
R/R: Relapsed/refractory
ORR: Overall response rate
CR: Complete remission
PFS: Progression-free survival
OS: Overall survival
