# Supplementary material for: Predictive factors and outcomes for ibrutinib in relapsed/refractory marginal zone lymphoma: a multicenter cohort study
Source: J Hematol Oncol. 2022 Jul 16;15:96. doi: 10.1186/s13045-022-01316-1 (PMC9287914; doi:10.1186/s13045-022-01316-1)
Supplement: Supplementary file 1 — Additional file 1. Supplemental Appendix. [file 13045_2022_1316_MOESM1_ESM.docx]

**SUPPLEMENTAL APPENDIX**

**Table of Contents** 1

**Methods**  2-3

**Results**  3

**Factors predictive of PFS in R/R/ MZL on ibrutinib:** Table S1 4

**Factors predictive of OS in R/R/ MZL on ibrutinib:** Table S2 5

**Impact of prior line of therapy on ibrutinib outcomes:** Table S3 6

**Duration of response:** Figure S1 7

**Survival outcomes in R/R MZL treated with ibrutinib:** Figure S2 8

**Survival outcomes based on the ibrutinib line of therapy:** Figure S3 9

**Survival outcomes based on prior line of therapy (IB in second line):** Figure S4 10

**Survival outcomes based on prior line of therapy (IB in third line):** Figure S5 11

**METHODS**

**Patients**

The study was approved by the institutional review boards at all the participating sites and performed in compliance with the Declaration of Helsinki. To be eligible for the analysis, patients must have received ibrutinib in the relapsed/refractory setting (at least one line of prior systemic therapy).

**Study definitions**

Primary refractory disease was defined as patients who had no response or progression of disease at the end of first-line therapy or within 6 months of treatment completion. DOR was defined as the interval from the first objective response (when either CR or PR was first determined) to progression or death, whichever occurred first. PFS was defined as the time from the start of ibrutinib therapy until lymphoma relapse/progression or death from any cause, censoring at the last clinical assessment if no progression or death. OS was defined as the time from the start of ibrutinib treatment until death or last follow‐up. There was no central imaging review and tumor assessment was performed accordingly to individual center practices. Four weeks of ibrutinib treatment constituted one cycle of ibrutinib.

**Statistical analysis**

Demographic and disease characteristics were summarized using descriptive statistics such as median and range for continuous variables, and frequency and percentage for categorical variables, compared among study groups using the Kruskal-Wallis test or Fisher’s exact test. The associations between patient characteristics and response to ibrutinib (CR/PR, SD, PP) were evaluated using the multinomial regression model with CR/PR as the base outcome. PFS and OS were estimated using the Kaplan-Meier method and compared between groups via the Log-rank test. Cox proportional hazard regression models were used to estimate the hazard ratios for risk of progression or death. Analyses were performed using Stata software (version 16; StataCorp, College Station, Texas), all statistical tests were two-sided with a type-1 error of 0.05.

**RESULTS**

**Patient characteristics**

The median age at the start of ibrutinib therapy was 68 years (range, 27-91 years) with 54% women. The majority of patients had stage 3-4 disease (83%) and an ECOG PS of 0-1 (94%). Nodal MZL was the most common subtype (n=50, 42%) followed by extranodal MZL (n=40, 34%), and SMZL (n=29, 24%). Among the patients who had bone marrow involvement at diagnosis (n=67), only 15% (n=10) had complex cytogenetics. The most commonly used first-line therapy was rituximab (49%) followed by bendamustine and rituximab (BR) (25%), rituximab, cyclophosphamide, vincristine, prednisone (R-CVP, 9%), rituximab, cyclophosphamide, adriamycin, vincristine, prednisone (R-CHOP, 8%). The median follow-up for the entire group was 23 months (range, 1-75 months) from initiation of ibrutinib. See Table 1.

**Histologic transformation**
There were a total of 6 patients (5%) who had histologic transformation in the study. The breakdown among the response groups was as follows: 3 patients (4%) in the CR/PR group, 2 patients (6%) in the SD group, and 1 (7%) in the PP group (p=0.85).

**Table S1. Factors predictive of PFS in patients with R/R MZL on ibrutinib**

| **Variable** | **HR** | **95% CI** | **p-value** |
| --- | --- | --- | --- |
| Age at ibrutinib therapy | 1.03 | 0.99-1.08 | 0.10 |
| Gender |  |  |  |
| Male | Referent |  |  |
| Female | 1.01 | 0.59-1.73 | 0.97 |
| BMI |  |  |  |
| <30 | Referent |  |  |
| ≥30 | 0.89 | 0.43-1.82 | 0.75 |
| ECOG PS at diagnosis |  |  |  |
| 0 | Referent |  |  |
| 1 | 0.80 | 0.44-1.45 | 0.46 |
| ≥2 | 0.93 | 0.28-3.09 | 0.90 |
| MZL subtype |  |  |  |
| NMZL | Referent |  |  |
| SMZL | 0.91 | 0.47-1.76 | 0.78 |
| EMZL | 0.88 | 0.46-1.65 | 0.68 |
| Stage at diagnosis |  |  |  |
| 1-2 | Referent |  |  |
| 3-4 | 0.86 | 0.42-1.77 | 0.68 |
| B symptoms at diagnosis |  |  |  |
| No | Referent |  |  |
| Yes | 1.18 | 0.65-2.15 | 0.59 |
| LDH higher than institutional baseline |  |  |  |
| No | Referent |  |  |
| Yes | 1.27 | 0.67-2.41 | 0.46 |
| Monoclonal protein at diagnosis |  |  |  |
| No | Referent |  |  |
| Yes | 0.84 | 0.44-1.63 | 0.61 |
| BM involvement at diagnosis |  |  |  |
| No | Referent |  |  |
| Yes | 1.17 | 0.59-2.33 | 0.66 |
| TP53 mutation/17p deletion |  |  |  |
| No | Referent |  |  |
| Yes | 1.19 | 0.41-3.45 | 0.74 |
| Complex cytogenetics |  |  |  |
| No | Referent |  |  |
| Yes | 3.08 | 1.23-7.67 | **0.02** |
| Primary refractory disease |  |  |  |
| No | Referent |  |  |
| Yes | 1.46 | 0.82-2.63 | 0.20 |
| Line of ibrutinib therapy |  |  |  |
| Second line | Referent |  |  |
| Third line | 1.00 | 0.54-1.86 | 0.99 |
| Fourth line and beyond | 1.17 | 0.58-2.36 | 0.65 |

Abbreviations: CR-complete response, PR-partial response, SD-stable disease, PD-progressive disease, BMI-body mass index, ECOG PS-Eastern Cooperative Oncology Group performance status, MZL- marginal zone lymphoma, LDH-lactate dehydrogenase, BM-bone marrow

**Table S2. Factors predictive of OS in patients with R/R MZL on ibrutinib**

| **Variable** | **HR** | **95% CI** | **p-value** |
| --- | --- | --- | --- |
| Age at ibrutinib therapy | 0.98 | 0.96-1.01 | 0.15 |
| Gender |  |  |  |
| Male | Referent |  |  |
| Female | 0.73 | 0.34-1.59 | 0.43 |
| BMI |  |  |  |
| <30 | Referent |  |  |
| ≥30 | 1.27 | 0.48-3.33 | 0.63 |
| ECOG PS at diagnosis |  |  |  |
| 0 | Referent |  |  |
| 1 | 1.54 | 0.60-3.97 | 0.37 |
| ≥2 | 3.02 | 0.77-11.78 | 0.11 |
| MZL subtype |  |  |  |
| NMZL | Referent |  |  |
| SMZL | 1.05 | 0.42-2.63 | 0.92 |
| EMZL | 0.94 | 0.36-2.42 | 0.89 |
| Stage at diagnosis |  |  |  |
| 1-2 | Referent |  |  |
| 3-4 | 0.88 | 0.30-2.59 | 0.81 |
| B symptoms at diagnosis |  |  |  |
| No | Referent |  |  |
| Yes | 1.72 | 0.74-3.98 | 0.20 |
| LDH higher than institutional baseline |  |  |  |
| No | Referent |  |  |
| Yes | 2.09 | 0.83-5.29 | 0.12 |
| Monoclonal protein at diagnosis |  |  |  |
| No | Referent |  |  |
| Yes | 0.77 | 0.29-2.03 | 0.60 |
| BM involvement at diagnosis |  |  |  |
| No | Referent |  |  |
| Yes | 1.67 | 0.56-4.98 | 0.36 |
| TP53 mutation/17p deletion |  |  |  |
| No | Referent |  |  |
| Yes | 2.07 | 0.57-7.50 | 0.27 |
| Complex cytogenetics |  |  |  |
| No | Referent |  |  |
| Yes | 3.00 | 1.03-8.68 | **0.04** |
| Primary refractory disease |  |  |  |
| No | Referent |  |  |
| Yes | 1.64 | 0.73-3.72 | 0.23 |
| Line of ibrutinib therapy |  |  |  |
| Second line | Referent |  |  |
| Third line | 0.91 | 0.35-2.32 | 0.84 |
| Fourth line and beyond | 1.73 | 0.68-4.42 | 0.25 |
| Response to IB |  |  |  |
| CR/PR | Referent |  |  |
| SD | 2.35 | 0.91-6.10 | 0.08 |
| PP | 13.94 | 5.17-37.62 | **<0.001** |

Abbreviations: CR-complete response, PR-partial response, SD-stable disease, PD-progressive disease, BMI-body mass index, ECOG PS-Eastern Cooperative Oncology Group performance status, MZL- marginal zone lymphoma, LDH-lactate dehydrogenase, BM-bone marrow

**Table S3. Impact of prior line of therapy on ibrutinib outcomes in patients with R/R MZL**

**Table S3A: Outcomes among 54 patients treated with ibrutinib in second line based on the frontline therapy**

|  | **Frontline treatment** | | | |
| --- | --- | --- | --- | --- |
| **Ibrutinib response** | **Rituximab**  **(n=26)** | **BR**  **(n=17)** | **R-CHOP/R-CVP**  **(n=8)** | **Others**  **(n=3)** |
| CR/PR | 15 (58) | 9 (53) | 5 (63) | 2 (67) |
| SD | 8 (31) | 6 (35) | 1 (12) | 1 (33) |
| PD* | 3 (11) | 2 (12) | 2 (25) | 0 (0) |

Abbreviations: CR-complete response, PR-partial response, SD-stable disease, PD-progressive disease, BR-bendamustine rituximab, R-CVP-rituximab, cyclophosphamide, vincristine, prednisone, R-CHOP-rituximab, cyclophosphamide, adriamycin, vincristine, prednisone

*Among the patients who had PP (n=7), 43% (n=3) received R in the frontline setting.

**Table S3B: Outcomes among 41 patients treated with ibrutinib in third line based on the second line therapy**

|  | **Second line treatment** | | | |
| --- | --- | --- | --- | --- |
| **Ibrutinib response** | **Rituximab**  **(n=15)** | **BR**  **(n=14)** | **PI3Ki**  **(n=2)** | **Others**  **(n=10)** |
| CR/PR | 10 (67) | 10 (53) | 1 (50) | 6 (60) |
| SD | 4 (27) | 4 (35) | 0(0) | 1 (10) |
| PD | 1 (7) | 0 (0) | 1 (50) | 3 (30) |

Abbreviations: CR-complete response, PR-partial response, SD-stable disease, PD-progressive disease, BR-bendamustine rituximab, PI3Ki

**Figure S1: Duration of response A) among all patients who achieved a response (CR/PR) B) based on CR or PR**


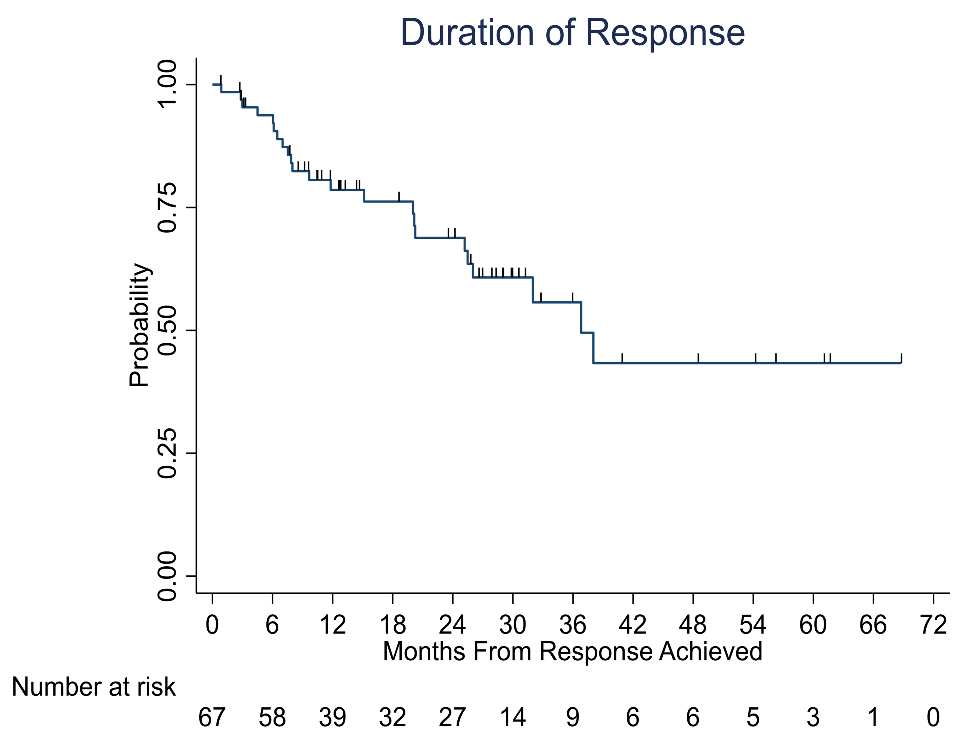

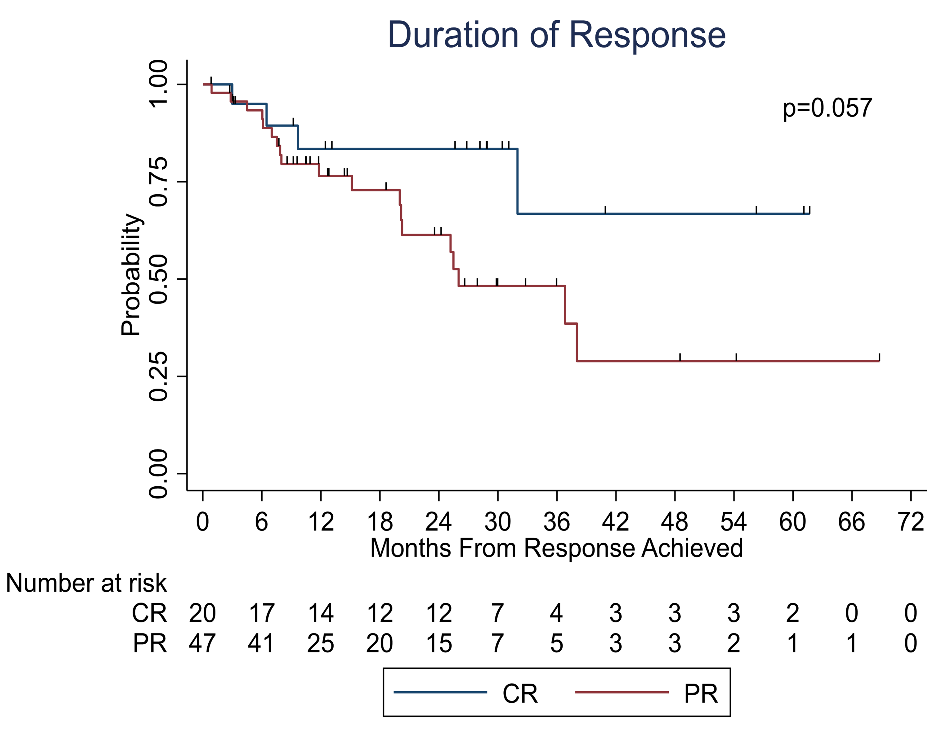


**Figure S2. Survival outcomes A) PFS and B) OS of patients with R/R MZL treated with ibrutinib**


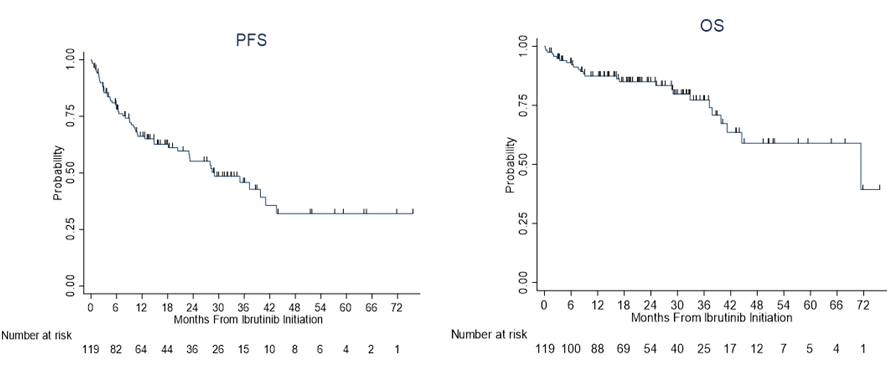


**Figure S3. Survival outcomes A) PFS and B) OS of patients with R/R MZL based on the ibrutinib line of therapy**


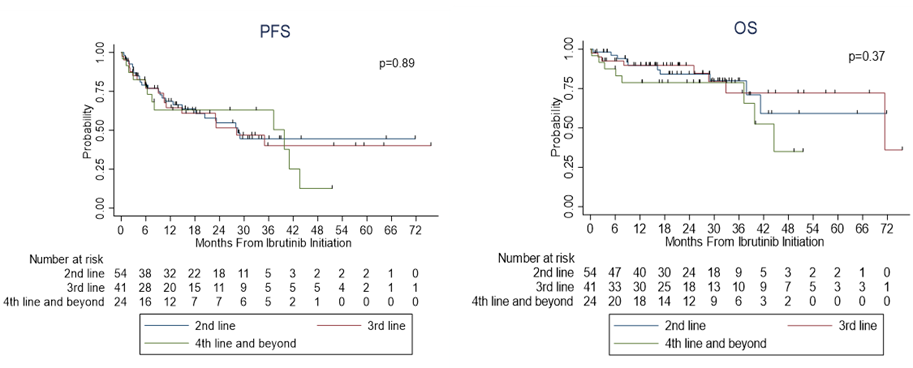


**Figure S4: Survival outcomes A) PFS and B) OS based on prior line of therapy (ibrutinib in second line)**


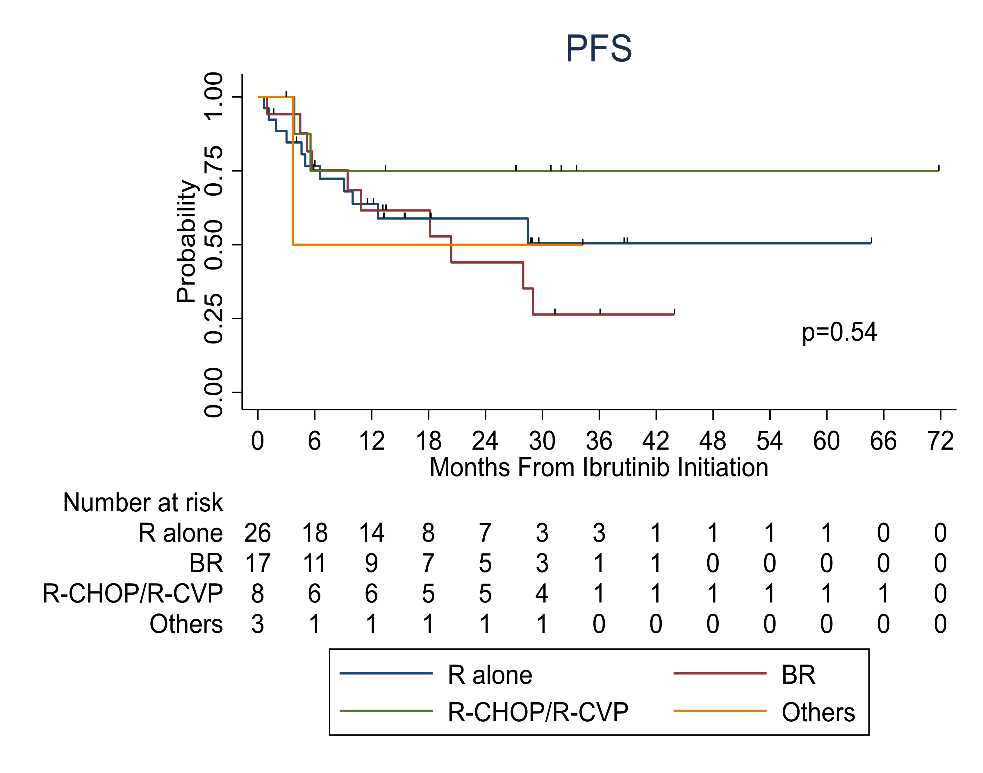

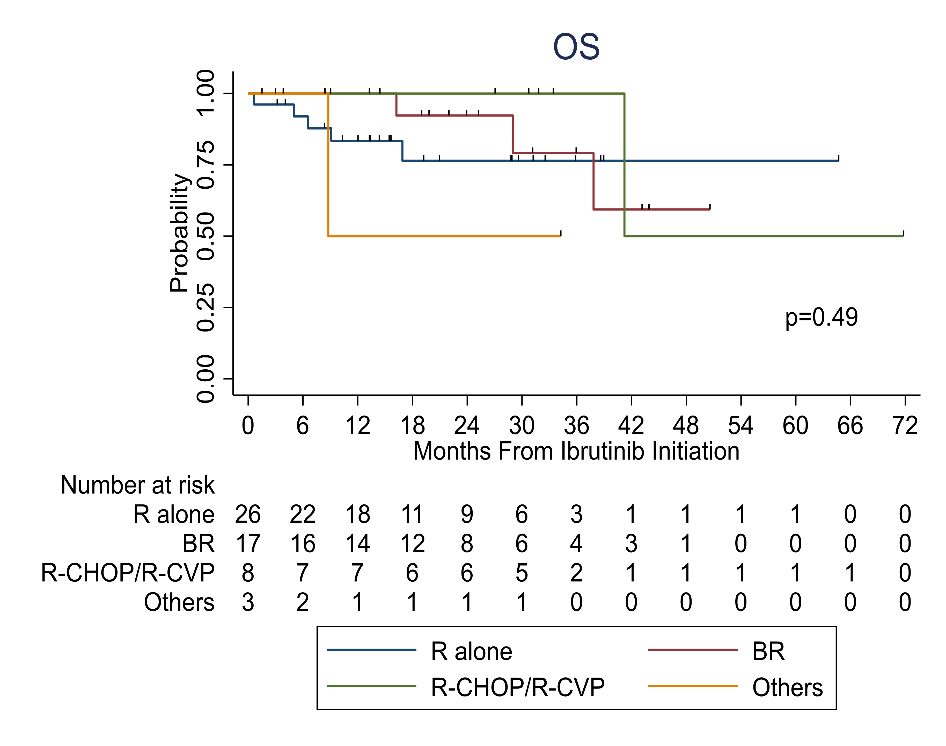


**Figure S5: Survival outcomes A) PFS and B) OS based on prior line of therapy (ibrutinib in third line)**

**
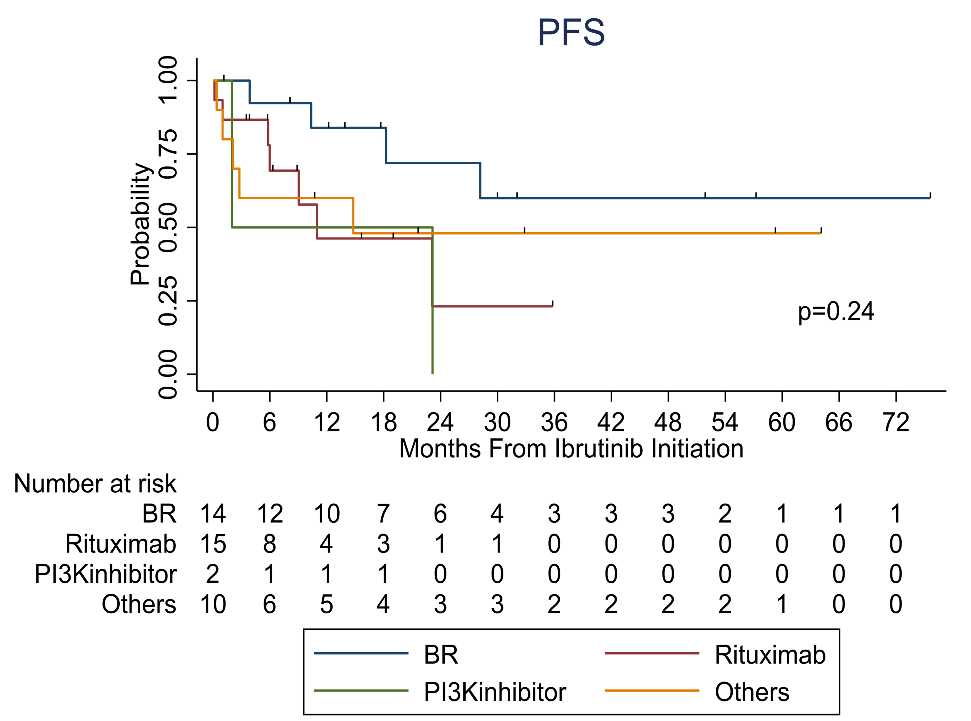

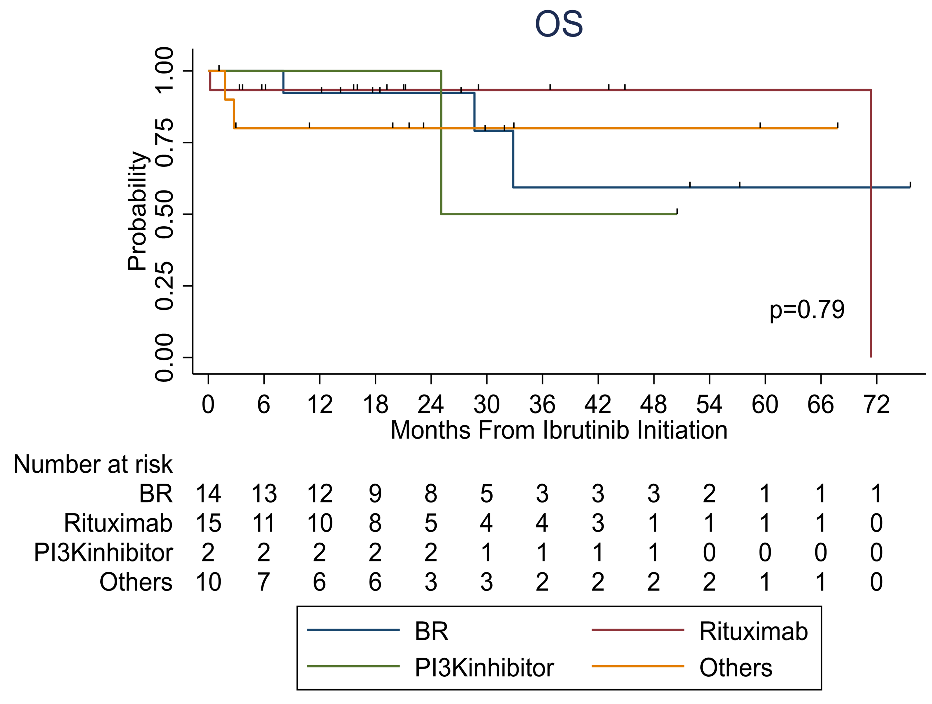
**
